# Phytochemical studies and antioxidant activity of two South African medicinal plants traditionally used for the management of opportunistic fungal infections in HIV/AIDS patients

**DOI:** 10.1186/1472-6882-12-43

**Published:** 2012-04-13

**Authors:** Wilfred Mbeng Otang, Donald Scott Grierson, Roland Ndip Ndip

**Affiliations:** 1 Department of Botany, School of Biological and Environmental Sciences, Faculty of Science and Agriculture, University of Fort Hare, P/Bag X1314, Alice 5700, South Africa; 2 Department of Biochemistry and Microbiology, School of Biological and Environmental Sciences, Faculty of Science and Agriculture, University of Fort Hare, P/Bag X1314, Alice 5700, South Africa; 3 Department of Microbiology and Parasitology, Faculty of Science, University of Buea, Box 63, Buea, Cameroon

**Keywords:** Antioxidant, phytochemical, *P. viridiflorum*, *G. bicolor*, Opportunistic fungi, HIV/AIDS

## Abstract

**Background:**

It has been observed that perturbations in the antioxidant defense systems, and consequently redox imbalance, are present in many tissues of HIV-infected patients. Hence, the exogenous supply of antioxidants, as natural compounds that scavenge free radicals, might represent an important additional strategy for the treatment of HIV infection. The aim of this study was therefore to analyse the phytochemical constituents and antioxidant potential of *Gasteria bicolor *Haw and *Pittosporum viridiflorum *Sims., two South African plants traditionally used for the management of opportunistic fungal infections (OFIs) in AIDS patients.

**Methods:**

The *in vitro *antioxidant properties of the two plants were screened through DPPH (1,1-diphenyl-2-picrylhydrazyl), NO (nitric oxide), H_2_O_2 _(hydrogen peroxide) radical scavenging effects and reducing power assays. Phytochemical studies were done by spectrophotometric techniques.

**Results:**

There were no significant differences in the flavonoid and proanthocyanidins contents between the leaves and bark extracts of *Gasteria bicolor *and *Pittosporum viridiflorum *respectively, while the total phenolic content of the bark extract of *P. viridiflorum *was significantly higher than that of *G. bicolor *leaf. The acetone extracts of both plants indicated strong antioxidant activities.

**Conclusion:**

The results from this study indicate that the leaves and stem extracts of *Gasteria bicolor *and *Pittosporum viridiflorum *respectively possess antioxidant properties and could serve as free radical inhibitors, acting possibly as primary antioxidants. Since reactive oxygen species are thought to be associated with the pathogenesis of AIDS, and HIV-infected individuals often have impaired antioxidant defenses, the inhibitory effect of the extracts on free radicals may partially justify the traditional use of these plants in the management of OFIs in HIV patients in South Africa.

## Background

HIV infection induces a wide array of immunologic alterations resulting in the progressive development of opportunistic infections and malignancy, which results in AIDS [1]. It has been observed that perturbations in the antioxidant defense systems, and consequently redox imbalance, are present in many tissues of HIV-infected patients [2]. Moreover, there is clear evidence that oxidative stress may contribute to several aspects of HIV disease, including viral replication, inflammatory response and decreased immune cell proliferation [1]. Hence, the exogenous supply of antioxidants, as natural compounds that scavenge free radicals, might represent an important additional strategy for the treatment of HIV infection.

Plants contain a wide variety of free radical scavenging molecules, such as flavonoids, anthocyanins, cartenoids, dietary glutathionine, alkaloids, tannins, saponins, steroids, terpenoids and rotenoids which are rich in antioxidant activities [1]. In South Africa, there is a rapid proliferation of the consumption of plant based decoctions by HIV infected people. Herbal preparations are cheap and simple; hence they remain a hope for the infected people who cannot access the government sponsored antiretroviral programmes [3].

In the Eastern Cape Province of South Africa, traditional healing practices often coexist with formalized and institutionalized medicine systems. Ethnobotanical surveys conducted in this region highlighted some plants used for the treatment of opportunistic fungal infections (OFIs) in HIV/AIDS such as the decoction of *Alepidea amatymbica *(Apiaceae) for aspergillosis, infusion of *Pittosporum viridiflorum *(Pittosporaceae) for cryptococcal meningitis, decoction of *Artemisia afra *(Asteraceae) for oesopharyngeal candidiasis, infusion of *Carpobrotus edulis *(Mesembyanthemaceae) for oral candidiasis, lotion of *Aloe ferox *(Liliaceae) for vaginal candidiasis, infusion of *Arctotis arctotoides *(Asteraceae) and lotion of *Gasteria bicolor *(Asphodelaceae) for dermatophytoses (Otang et al., manuscript accepted for publication).

The stem and leaves of *Gasteria bicolor *were examined [4]; three new dihydroanthracenones namely 3,4-dihydro-2,6,9-trihydroxy-8-methyl-1(2 *H*)-anthracenone (gasteriacenone A), 3,4-dihydro-2,4,9-trihydroxy-6-methoxy-8-methyl-1(2 *H*)-anthracenone (gasteriacenone B) and 3,4-dihydro-4,6,9-trihydroxy-7-carbomethoxy-8-methyl-1(2 *H*)-anthracenone (gasteriacenone C) were determined. Their structures were elucidated by spectroscopic methods including 2D NMR techniques. A literature survey revealed that several species of the genus *Pittosporum *have been studied for their secondary metabolites. Triterpenoids and saponins were isolated from *P. tobira, P. undulatum, P. phylliraeoides, P. pentaurum*, and *P. viridiflorum *[5-8]. Bioassay guided purification of the ethanolic extract of the bark of New Caledonian *Pittosporum pancheri* Brongn. and Gris (Pittosporaceae) led to the isolation and characterisation of two new farnesylmonoglycosides, pancherins A and B. The *in vitro *antifungal activity of *G. bicolor *and *P. viridiflorum *have been investigated against a panel of opportunistic fungi in HIV/AIDS in our previous study [9] and by other authors [7,10].

Although the antioxidant and phytochemical properties for some of these plants have been investigated in different countries [11-15], there is a dearth of knowledge of such studies on *G. bicolor *and *P. viridiflorum *in South Africa. The aim of this study was therefore to analyse the phytochemical constituents and antioxidant potential of these plants. The antioxidant potential of the extracts, determined by DPPH, NO, and H_2_O_2 _and reducing power assays are designated by their IC_50 _(concentration required to attain 50% radical-scavenging effect) and compared with that of the standards (rutin, vitamin C, and Butylatedhydroxytoluene). The phytochemical and antioxidant screening of these plants is a prerequisite for verification and utilisation as new sources of herbal drugs [16].

## Materials and methods

### General

Aluminium chloride (AlCl_3_), Gallic acid, Folin-Ciocalteu's phenol reagent, Sodium carbonate (Na_2_CO_3_) Sodium nitrite (NaNO_2_), 1,1-Diphenyl-2-picrylhydrazyl (DPPH), quercetin, trichloracetic acid (TCA), potassium ferricyanide, butylatedhydroxytoluene (BHT), vitamin C, tannic acid, Iron III chloride (FeCl_3_) were all purchased from Merck (South Africa). All chemicals and solvents used in this experiment were of analytical grade. Identification of the collected plants specimens was done by Prof. D.S. Grierson in the Department of Botany at the University of Fort Hare, South Africa. Voucher specimens with their corresponding numbers (W9 for *P. viridiflorum *and W28 for W31 for *G. bicolor*) were deposited in the Griffin Herbarium of the University of Fort Hare.

### Extraction procedure

Leaf and bark samples of *G. bicolor *and *P. viridiflorum *respectively were collected from locations around Alice, in the Eastern Cape Province of South Africa. The samples were dried at 48°C for 48 hours in an oven. Ground samples were percolated with acetone in the ratio 1:5 (w/v) at room temperature for 24 hours after which the extracts were decanted, filtered with Whatman No. 1 filter paper and concentrated in vacuo below 48°C to give the crude extracts used for the investigation [17].

### Phytochemical analysis

#### Determination of total phenolics

Total phenolic contents were evaluated with Folin-Ciocalteu's phenol reagent [18,19]. 5 ml of the extract solution was mixed with 5 ml Folin-Ciocalteu reagent previously diluted with water (1:9 v/v). After 5 minutes, 4 ml of 7% Na_2_CO_3 _solution was added with mixing. The tubes were vortexed for 5 seconds and allowed to stand for 30 min at 40°C for color development. Absorbance was then measured at 765 nm using the Hewlett Packard UV-vis spectrophotometer. Samples of extract were evaluated at a final concentration of 0.1 mg/ml. Total phenolic content was expressed as mg/g tannic acid equivalent using the following equation based on the calibration curve: y = 0.1216x, R^2 ^= 0.9365, where y was the absorbance x was the concentration.

#### Determination of total flavonoids

Colorimetric aluminum chloride method was used for flavonoid determination [17,19]. 0.5 ml solution of each plant extract in methanol was separately mixed with 0.5 ml of 2% aluminum chloride. After one hour at room temperature, the absorbance was measured at 420 nm. A yellow color indicated the presence of flavonoids. Extract samples were evaluated at a final concentration of 0.1 mg/ml. Total flavonoid content were calculated as quercetin equivalents (mg/g) using the following equation based on the calibration curve: y = 0.0255x, R^2 ^= 0.9812, where y was the absorbance x was the concentration.

#### Determination of total proanthocyanidins

Determination of proanthocyanidins was based on the standard procedures [20]. 0.5 ml of 1 mg/ml of extract solution was mixed with 3 ml of 4% vanillin-methanol solution (4% v/v) and 1.5 ml of hydrochloric acid was added and vortexed. The mixture was allowed to stand for 15 minutes at room temperature. The absorbance was then measured at 500 nm. Extract samples were evaluated at a final concentration of 0.1 mg/ml. Total proanthocyanidin contents were expressed as catechin equivalents (mg/g) using the following equation based on the calibration curve: y = 0.5825x, R^2 ^= 0.9277, where y was the absorbance x was the concentration.

#### Saponin determination

The saponin content in the plant extracts was estimated as previously described [21]. Ten grams of the powdered sample was placed in 200 ml of 20% ethanol. The suspension was heated in a water bath at 55°C for 4 hours with continuous stirring. The mixture was filtered and the residue was re-extracted as above. The combined extracts were reduced to 40 ml over a water bath at 90°C. The concentrate was transferred into a 250 ml separator funnel and 20 ml diethyl ether was added and shaken vigorously. The ether layer was discarded, while the purification process was repeated. 60 ml of n-butanol was added and the extracts were washed twice with 10 ml of 5% aqueous sodium chloride. The remaining solution was heated in a water bath. After evapoaration, the sample was dried in the oven to a constant weight. The saponin content was calculated according to the equation: amount of saponin (mg/g) = weight of residue/weight of sample.

#### Alkaloids determination

Five grams of the powdered sample was weighed into 200 ml of 20% acetic acid in ethanol and allowed to stand for 4 hours. This was filtered and the extract was concentrated using a water bath at 55°C to one-quarter of the original volume. Concentrated ammonium hydroxide was added drop wise into the extract until precipitation was complete. The whole solution was allowed to settle and the precipitate collected was washed with dilute ammonium hydroxide solution and then filtered. The residue which is the crude alkaloid was weighed and calculated according to the equation: amount of alkaloid (mg/g) = weight of precipitate/weight of sample [22].

### Antioxidant assays

#### Assay of DPPH scavenging activity

The DPPH radical-scavenging activity of the test extracts was examined as previously described [17]. Different concentrations (0.025 - 0.5 μg/ml) of each extract were added, at an equal volume, to methanolic solution of DPPH (100 μM). The mixture was allowed to react at room temperature in the dark for 30 minutes. Vitamin C and rutin were used as standard controls. Three replicates were made for each test sample. After 30 minutes, the absorbance (A) was measured at 518 nm and converted into the percentage antioxidant activity using the following equation: % scavenged [[]]. IC_50 _values denote the concentration of sample which is required to scavenge 50% of DPPH free radicals. The IC_50 _values were calculated by linear regression of plots, where the abscissa represented the concentration of the tested plant extracts and the ordinate the average percent of scavenging capacity from three replicates.

#### Assay of nitric oxide-scavenging activity

This assay was based according to the following procedure [17]. 2 ml of 10 mM sodium nitroprusside in 0.5 mM phosphate-buffered saline (pH 7.4) was mixed with different concentrations of each extracts dissolved in water and incubated at 25°C for 2.5 hours. After the incubation period, 0.5 ml of Griess reagent was added and the absorbance was read at 540 nm. Vitamin C and rutin were used as positive controls.

#### Reducing power assay

The reducing power of the plant extracts was determined according to the following method [17]. Different amounts of each extracts (0.025 - 0.05 μg/ml) in distilled water were mixed with 2.5 ml of 0.2 M phosphate buffer (pH 6.6) and 2.5 ml potassium ferricyanide (1% w/v). The resulting mixture was incubated at 50°C for 20 minutes, followed by the addition of 2.5 ml of trichloroacetic acid (10% w/v). This was then centrifuged at 3000 rpm for 10 minutes. 2.5 ml of the supernatant was mixed with an equal volume of distilled water and 0.5 ml of FeCl_3 _(0.1% w/v) and the absorbance was measured at 700 nm. Vitamin C and BHT were used as positive controls.

#### Scavenging of hydrogen peroxide

The ability of the extracts to scavenge hydrogen peroxide was determined according to standard procedures [23]. A solution of hydrogen peroxide (40 mM) was prepared in phosphate buffer (pH 7.4). Different concentrations of the plant extracts (0.025 - 0.05 μg/ml) in distilled water were added to a hydrogen peroxide solution (0.6 ml, 40 mM). The absorbance of hydrogen peroxide at 230 nm was determined after ten minutes against a blank solution containing phosphate buffer without hydrogen peroxide. The percentage of hydrogen peroxide scavenging by the extracts and standard compounds was calculated as follows: % Scavenged [H_2_O_2_] = [(Ao - A_1_)/Ao] × 100 where Ao was the absorbance of the control and A_1 _was the absorbance in the presence of the sample of extract and standard.

### Statistical analysis

All experiments were done in triplicates and where applicable, the data were subjected to one way analysis of variance (ANOVA) and differences between samples were determined by Duncan's Multiple Range test using the Minitab program (version 12 for windows). P Values < 0.05 were regarded as significant.

## Results

### Phytochemical analysis

#### Total phenolic, flavonoids and proanthocyanidin contents

**Figure 1:**
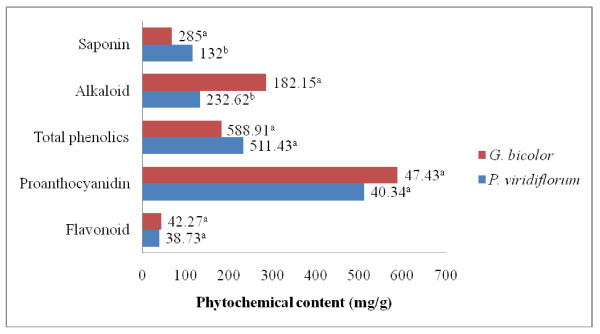
**Phytochemical constituents identified in the acetone extracts of *G. bicolor *(leaf) and *P. viridiflorum *(bark)**. Values with the same letter superscript within the same constituent are not significantly different (P > 0.05).

The level of these phenolic compounds in the acetone extracts of the leaves and stem bark of *G. bicolor *and *P. viridiflorum *respectively were considerable and are shown in Figure 1. The total phenolic content of the stem bark extract of *P. viridiflorum *bark was significantly higher (P < 0.05) than that of *G. bicolor *leaf (Figure 1). There was no significant difference (P > 0.05) in the total flavonoid and proanthocyanidins contents between the leaves and stem bark extracts of *G. bicolor *and *P. viridiflorum *respectively (Figure 1).

#### Alkaloid and saponin content

Quantitative estimation indicated that the alkaloid content in the leaf extract of *G. bicolor *(285.0 mg/g) was significantly higher (P < 0.05) than that of *P. viridiflorum *bark (Figure 1). On the other hand, the saponin content of the bark of *P. viridiflorum *was significantly higher (P < 0.05) than that of *G. bicolor *leaves.

### Antioxidant assay

#### DPPH radical-scavenging activity

**Figure 2:**
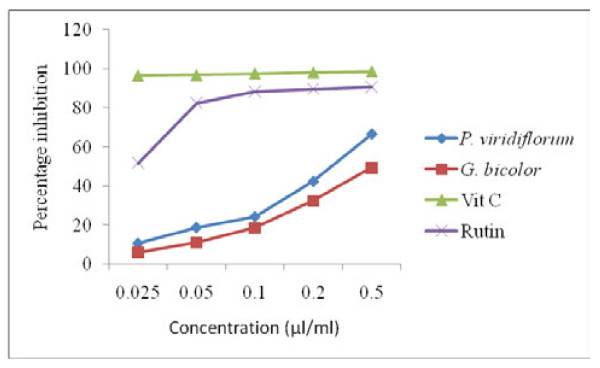
**DPPH scavenging activity of *G. bicolor *(leaf) and *P. viridiflorum *(bark) extracts**. Results are means of 3 replicates.

The concentration required to attain 50% radical-scavenging effect (IC_50_) was determined from the results of a series of concentrations tested. A lower IC_50 _value corresponds to a larger scavenging activity [24]. The IC_50 _values of the tested samples were in the order: vitamin C < rutin <*P. viridiflorum *<*G. bicolor *(Table 1). Scavenging activity was expressed as percentage of inhibition of DPPH free radical (Figure 2). The results of the DPPH assay also showed that *P. viridiflorum *bark extract has a stronger scavenging activity than that of *G. bicolor *leaves at all concentrations. The percentage inhibition of DPPH radical by *P. viridiflorum *extract was 66.7% at 0.05 μg/ml. As shown in Figure 2, the DPPH radical-scavenging activity of the acetone extracts of *P. viridiflorum and G. bicolor *and the standards (vitamin C and rutin) was shown to occur in a dose-dependent manner. However, none of the plant samples evaluated here showed an activity that was as strong as that of vitamin C.

**Table 1:** Scavenging activity of *P. viridiflorum *(bark) and *G. bicolor *(leaf) acetone extracts

Sample	DPPH		Nitric oxide		Reducing power		Hydrogen peroxide	
	
	**IC_50_**^a^	R^2 b^	IC_50_	R^2^	IC_50_	R^2^	IC_50_	R^2^
*P. viridiflorum*	0.22	95.70	0.16	39.10	0.26	83.20	0.13	89.90
*G. bicolor*	0.27	91.80	0.22	93.60	0.27	99.90	0.23	92.20
Vitamin C	0.05	73.90	0.24	96.10	0.22	92.40	0.29	92.50
Rutin	0.08	30.50	0.25	99.30	-	-	-	-
BHT	-	-	-	-	0.23	95.90	-	-
Gallic acid	-	-	-	-	-	-	0.15	55.90

a: IC_50 _is defined as the concentration (μg/ml) sufficient to obtain 50% of a maximum scavenging capacity.

b: coefficient of determination; values obtained from regression lines with 95% confidence level.

-: Values not determined

#### Nitric oxide scavenging activity

**Figure 3:**
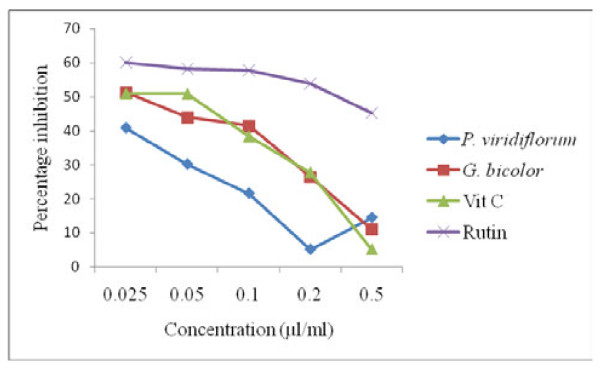
**Nitric oxide scavenging activity of *G. bicolor *(leaf) and *P. viridiflorum *(bark) extracts**. Results are means of 3 replicates.

The acetone extract of *P. viridiflorum *showed a concentration dependent decrease in NO scavenging activity that reached a minimum at a concentration of 0.2 μg/ml and increased thereafter (Figure 3). Both *G. bicolor *extract and vitamin C also showed a dose dependent decrease in NO scavenging activity which was lower than that of rutin at all the tested concentrations.

#### Reducing power assay

**Figure 4:**
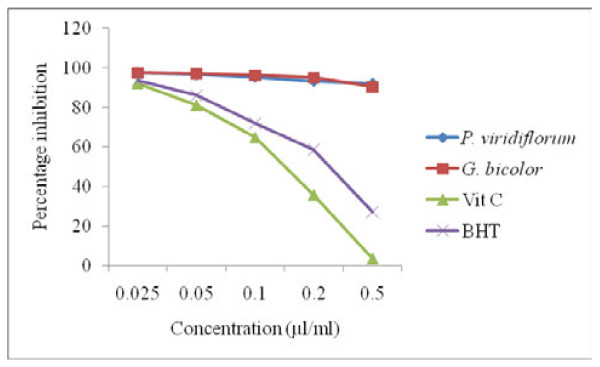
**Reducing power of *G. bicolor *(leaf) and *P. viridiflorum *(bark) extracts**. Results are means of 3 replicates.

The dose-response curves for the reducing powers of the tested samples (as indicated by the absorbance at 700 nm) are shown in Figure 4. Increased absorbance of the reaction mixture indicated increased reducing power [17]. The reducing power of both the crude extracts and standards which correlated well with their concentrations was in the order: vitamin C < BHT <*P. viridiflorum < G. bicolor *at a concentration of 0.2 μg/ml. The reducing ability of both plant extracts was significantly greater than that of vitamin C (P < 0.05).

#### Hydrogen peroxide scavenging activity

**Figure 5:**
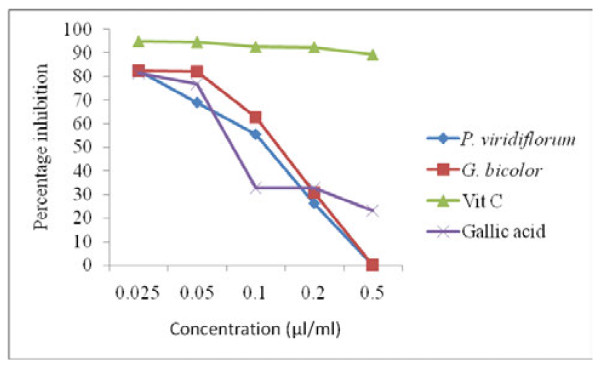
**H_2_O_2 _scavenging activity f *G. bicolor *(leaf) and *P. viridiflorum *(bark) extracts**. Results are means of 3 replicates.

The IC_50 _for scavenging of H_2_O_2 _ranges from 0.15 - 0.29 μl/ml (Table 1). The H_2_O_2 _scavenging activity of both plant extracts and standards decreased with increasing concentration (Figure 5). The scavenging activity of *G. bicolor *was higher than that of the standard, gallic acid within the concentration range of 0.025 - 0.2 μl/ml.

## Discussion

The results obtained in the present study revealed that the level of the phenolic compounds in the acetone extracts of the leaves and stem bark of *G. bicolor *and *P. viridiflorum *respectively were considerable, with the total phenolic content of the stem bark extract of *P. viridiflorum *significantly higher (P < 0.05) than that of the leaf of *G. bicolor*. In a similar study, the methanol extract of *P. manni *(synonym; *viridiflorum*) derived from Cameroon was shown to be very rich in phenolic derivatives (having 314 ± 4 mg/g gallic acid equivalent). Evidence supports the premise that a pro-oxidant condition exists in HIV-seropositive patients, a result of an overabundance in the production of reactive oxygen forms combined with a multilevel deficiency in nutritional and metabolic sources of antioxidants [25]. This therefore, suggests that extracts of *P. viridiflorum *could be used as a source of natural antioxidants to limit free radical damage occurring in AIDS patients by acting in a synergistic manner and inhibit the destruction of cells by HIV.

Flavonoids reduce free radicals by quenching, up-regulating or protecting antioxidant defences and chelating radical intermediate compounds [26]. Alkaloids and their synthetic derivatives have been shown to have analgesic activities and they exhibit marked physiological activity when administered to animals [27]. Some of the characteristics of saponins include formation of foams in aqueous solutions, hemolytic activity, cholesterol binding properties and bitterness. It has also been shown that saponins are active antifungal agents [28]. These may partially justify the traditional use of *P. viridiflorum *and *G. bicolor *in the treatment of mycotic infections associated with HIV/AIDS.

The concentration required to attain 50% radical-scavenging effect (IC_50_) was determined from the results of a series of concentrations tested. A lower IC_50 _value corresponds to a larger scavenging activity and a DPPH radical scavenging activity of 50% and above is considered significant [23]. Hence, based on the IC_50 _values, *P. viridiflorum *bark extract exhibited a stronger scavenging activity than that of *G. bicolor *leaves at all concentrations. A similar study revealed that the methanol extract of *P. viridiflorum *(bark) in Cameroon showed an excellent inhibitory activity of 68.82% against DPPH radical at a concentration of 250 μg/ml [11]. These results indicate that *P. viridiflorum *could be an excellent source of natural antioxidants and deserves further investigation. The fact that none of the plant samples evaluated showed an activity that was as strong as that of vitamin C was attributed the fact that the additive or synergistic effects of polyphenols make the antioxidant activity of the extracts weaker than that of the isolated bioactive compounds [29]. In addition, the total phenolic content in the crude extracts does not incorporate all the antioxidants. The phenolic compounds present in the extracts could be responsible for the observed DPPH radical scavenging activity, since they can readily donate hydrogen atom to the radical [24].

Both *G. bicolor *extract and vitamin C also showed a dose dependent elevation in NO scavenging activity which was higher than that of rutin at all the tested concentrations. Generation of reactive nitrogen species beyond the capacity of a biological system to eliminate them gives rise to oxidative stress, which plays a role in the pathogenesis of opportunistic infections in HIV/AIDS, and natural antioxidants could be helpful in the management of such diseases mediated by oxidative stress [30]. The reducing power of both the crude extracts and standards which correlated well with their concentrations was in the order: vitamin C > BHT >*P. viridiflorum > G. bicolor *at a concentration of 0.2 μg/ml. Plant extracts with reducing properties have been shown to exert antioxidant action by breaking the free radical chain through the donation of a hydrogen atom [23], while the scavenging of H_2_O_2 _by the plant extracts was attributed to their phenolics, which can donate electrons to H_2_O_2_, thus neutralizing it to water [17]. Although hydrogen peroxide is not very reactive, it can sometimes cause cytotoxicity by giving rise to hydroxyl radicals in the cell, thus, removing H_2_O_2 _is very important [19].

## Conclusion

The presence of phytochemicals such as phenolics, alkaloids, flavonoids, saponins, proanthocyanidins in *G. bicolor *(leaf) and *P. viridiflorum *(bark) provides some scientific evidence for the traditional usage of these plants in the management of OFIs in HIV/AIDS patients. The crude acetone extracts of the plants have also indicated strong antioxidant activities *in vitro*. Since reactive oxygen species are thought to be associated with the pathogenesis of HIV/AIDS, and HIV-infected individuals have impaired antioxidant defenses, the inhibitory effect of the extracts on the free radicals may partially justify the traditional use of *P. viridiflorum *and *G. bicolor *in the management of opportunistic fungi by AIDS patients in the Eastern Cape Province of South Africa. Notwithstanding, further research is necessary to isolate the active components of these extracts.

## Abbreviations

AlCl_3_: Aluminium chloride; BHT: Butylatedhydroxytoluene; DPPH: 2,2-Diphenyl-1-Picrylhydrazyl; IC_50_: The effective concentration required to attain 50% radical-scavenging effect; FeCl_3_: Iron III chloride; H_2_O_2_: Hydrogen peroxide; NO: Nitric oxide; OFIs: Opportunistic fungal infections; Na_2_CO_3_: Sodium carbonate; NaNO_2_: Sodium nitrite; TCA: Trichloracetic acid.

## Competing interests

The authors declare that they have no competing interests.

## Authors' contributions

WM carried out the phytochemical and antioxidants laboratory experiments, and drafted the manuscript. DS participated in the laboratory investigations and design of the study. RN participated in the design and coordination. All authors read and approved the final manuscript.

## Pre-publication history

The pre-publication history for this paper can be accessed here:

http://www.biomedcentral.com/1472-6882/12/43/prepub
